# Association between central auditory processing mechanism and cardiac autonomic regulation

**DOI:** 10.1186/1755-7682-7-21

**Published:** 2014-05-07

**Authors:** Simone F Regaçone, Daiane DB Lima, Mariana S Banzato, Ana CB Gução, Vitor E Valenti, Ana CF Frizzo

**Affiliations:** 1 Departamento de Fonoaudiologia, Faculdade de Filosofia e Ciências, UNESP, Av. Hygino Muzzi Filho, 737. 17525-900 Marília, SP, Brasil

**Keywords:** Auditory stimulation, Autonomic nervous system, Heart rate, P300

## Abstract

**Background:**

This study was conducted to describe the association between central auditory processing mechanism and the cardiac autonomic regulation.

**Methods:**

It was researched papers on the topic addressed in this study considering the following data bases: Medline, Pubmed, Lilacs, Scopus and Cochrane. The key words were: “auditory stimulation, heart rate, autonomic nervous system and P300”.

**Results:**

The findings in the literature demonstrated that auditory stimulation influences the autonomic nervous system and has been used in conjunction with other methods. It is considered a promising step in the investigation of therapeutic procedures for rehabilitation and quality of life of several pathologies.

**Conclusion:**

The association between auditory stimulation and the level of the cardiac autonomic nervous system has received significant contributions in relation to musical stimuli.

## Background

Listen to several auditory stimulation requires the brain articulation of various tasks, it is an act designed to engage perception, cognition, emotion, memory and learning that goes through various stages of processing, causing reactions to reach conscious awareness. Thus, central auditory processing mechanism is a rich and indispensable tool in the investigation of the functioning of the nervous system [1].

In relation to musical auditory stimulation, since ancient time music had its importance to mankind, the Greeks had music as a divine gift, a concept for healing the body and mind in the figurative image of the God of medicine and music, Apollo. However, the song became valued in scientific circles recently, with noticeable benefits to pathological individuals [1].

Several scholars have mentioned the importance of integrating music in the rehabilitation phase, suggesting that this method brings beneficial results in relation to cognitive, emotional and social needs of individuals of all ages, prioritizing the quality of life of these subjects [2-6].

The literature shows the beneficial effects of music both in pre-surgical patients in relieving pain and anxiety [3], and in post-surgical phase, acting on the autonomic nervous system, reducing heart rate, blood pressure and pain [7].

It is known that the method used in scientific production in relation to the types of auditory stimulation parameters associating cardiovascular and autonomic nervous system has been the music [5]. On the other hand, it is very limited the use of other auditory stimuli in this situation.

In this context, an important method used to analysis the central auditory process is the potential long latency auditory, also called as P300 [8]. This method is used to assess functioning and integrity of the auditory pathway. Considering that the both cardiovascular [9,10] and auditory systems [8] are processed in the brain, it is surmised the relevance of studies focusing on this interaction, however, it lacks in the literature solid evidence of this interaction. Therefore, this study aimed to describe the association between central auditory processing mechanism and cardiac autonomic regulation.

## Methods

A literature review was conducted between September 2012 and March 2013 and it was divided into two stages: a survey of the material, classification and content analysis.

In the first stage the studies were surveyed on the topic addressed in this study, considering the following data bases: Medline, Pubmed, Lilacs, Scopus and Cochrane. Initially a search was made from the title and then collecting their summaries. The key words were: “auditory stimulation, heart rate and P300”. The descriptors used v according to the Medical Subject Headings (MeSH). All references were between 2003 and 2013.

The second stage was supervised by two reviewers, an expert in the field of audiology and the other in cardiac autonomic regulation, which included the classification and analysis of the abstracts, according to the focus of the study. We excluded studies which did not cover the thematic objective of the review, published after the deadline, with no scientific content, summaries, the same publication in various media, proceedings of seminars and conferences, thesis and dissertations, and studies that were not part of an indexed journal.

After this first analysis, the titles were selected to undergo a new assessment, with more specific criteria considering clinical trials, observational studies, and basic studies investigating cardiovascular parameters and auditory stimulation.

## Results

The electronic search yielded a total of 1500 references. Reading the titles and abstracts, and after the application of exclusion criteria, 102 articles emained. A complete review of the literature allowed the inclusion and analysis of 19 articles, considered of high scientific impact and according to the theme of the literature. Table 1 summarizes some of the studies and their main findings.

**Table 1:** Main studies regarding the effects of auditory stimulation on cardiac autonomic regulation

***Authors and year***	***Main conclusions***
Lee *et al*., 2010 [11]	White noise exposure over 50 dB increases sympathetic activity and there is strong correlation between LF/HF ratio and noise intensity.
Roque *et al*., 2013 [12]	The authors suggested that relaxant baroque and excitatory heavy metal music slightly decrease global heart rate variability because of the equivalent sound level.
Roque *et al*., 2013 [13]	It was indicated that acute exposure to heavy metal music affected the sympathetic activity in healthy women.
Nakamura *et al*., 2007 [14]	Music reduces renal sympathetic nerve activity and blood pressure through the auditory pathway, the hypothalamic suprachiasmatic nucleus, and histaminergic neurons.
De Castro *et al*., 2013 [15]	Pleasure in response to music induces dopamine release in the striatal system.
Kraus *et al.*, 2010 [16]	Music training presents responses similar to physical exercise and it is a resource that tones the brain for auditory fitness.
Sarkamo *et al.*, 2008 [17]	Musical auditory stimulation improved emotional and cognitive recovery in subjects with post-stroke.

LF/HF ratio: Low frequency/high frequency ratio; dB: decibel.

## Discussion

In this study review we described previous studies that investigated the influence of auditory stimulation on heart rate variability. According to the findings in the literature auditory stimulation influences the autonomic nervous system and has been used in conjunction with other methods. Regarding the types of auditory stimulation, music was the most used method. The analysis of texts selected for this review indicated that harmonic music is able to improve cardiac autonomic regulation. The literature on the effect of music on the autonomic nervous system activity in healthy subjects is quite large. Conversely, the literature on how musical auditory stimulation affects individuals with cardiovascular dysfunction is less developed.

A previous study cited in our review [15] suggested that music education during childhood, albeit limited, can influence the adult brain, reinforcing the idea that music is a positive agent of experience-dependent neural plasticity. The authors [15] evaluated the influence of music on physiological mechanisms in the human body, especially on blood pressure, as well as identifying the neural mechanisms of music processing. They concluded that music can present a real role in regulating blood pressure, heart rate and respiratory rate, and other benefits such as the reduction of anxiety and pain. A possible explanation for this mechanism is that it is related to the induction of emotional feelings [18]. The emotions induced by music are evoked by temporal phenomena, such as expectations, delay, tension, resolution, prediction, surprise and anticipation [18].

Two studies previously published [12,13] aimed to evaluate the effects of two music from different styles on heart rate variability. Interestingly, auditory stimulation with Pachelbel (Canon in D) reduced global heart rate variability through time and frequency domain and geometric indices. Heavy metal music with Gamma Ray (Heavy metal universe) also decreased the same indices. According to the literature, auditory stimulation with music influences the cardiovascular and autonomic function [11,18-21]. On the other hand, the authors hypothesized that the cardiac autonomic responses induced by music depends on the music style, since musical auditory stimulation is involved in emotional induction [18].

In another study [17] it was reported that patients with post-stroke after early auditory stimulation through music presented improvements in cognitive and emotional recovery. Listening to music was indicated to present beneficial effect on blood pressure, heart rate, respiratory rate, anxiety and pain in people with coronary heart disease [22]. In this sense, researchers have studied the effects of sound on the transplant cardiac graft autoimmune responses in an animal model. It was indicatde that exposure to opera music such as La Traviata could affect aspects of peripheral immune cells and the generation of regulatory CD4 + CD25 + cells and up-regulation of anti-inflammatory cytokines, resulting in prolonged graft survival [23]. Moreover, the use of music therapy in patients with surgical procedures in the postoperative period of cardiac surgery [24] showed a significant reduction of pain and anxiety, demonstrating that patients recovering from heart surgery can benefit from music therapy.

Although exposure to music can lower blood pressure and renal sympathetic nerve activity, it is important to reinforce that not all music styles have this effect, and that the effects of musical stimulation on the cardiovascular system depend on an intact cochlea and proper functioning of histaminergic neurons in the suprachiasmatic nucleus of the hypothalamus [14]. Furthermore, rats with bilateral lesions of the auditory cortex were able to discriminate a simple auditory stimulation, indicating that there is another auditory processing pathway that is not mediated by the auditory cortex [25].

Within this context, some authors suggested that music can broaden understanding of neuroplasticity in the long term, and that these findings have implications for policy makers and education for the development of auditory training programs that can generate lasting positive results [12]. In addition, other authors [26] believe that music therapy could be inserted into programs of multidisciplinary care for hypertensive population, due to its contribution to the control of blood pressure and improving the quality of life of these patients. However, the scientific evidence of those results are not yet strong, making several authors suggest more research on the effects of music applied by qualified music therapists [22].

Exploring the literature we found a study making association between the P300 wave (P300) and musical auditory stimulation in young women in order to avoid habituation [27]. The P300 wave is an event related potential component elicited in the process of decision making. It is considered to be an endogenous potential, as its occurrence links not to the physical attributes of a stimulus, but to a person's reaction to it [28]. The mentioned study [27] group was exposed to music at different preset speeds before the assessment of P300 and the control group was not exposed to musical stimulation. The findings of this research showed that the P300 amplitude was not affected in young research group observing sustained attention during testing, which was not observed in the control group. The authors concluded that musical stimulation can be useful in the retest P300 and avoid habituation. Nevertheless, studies with auditory stimulation other than music are rare in the literature.

Another study [29] to test simulated driving mental fatigue observed significant reduction in P300 amplitude, indicating that when the individual was occurring mental fatigue induced a decrease in attention to the auditory task. These results suggest that the physiological response is related to the auditory mental fatigue, which has an impact on the function of the central nervous system, which consequently controls and regulates the cardiovascular system.

We found in our literature review some studies [30-32] that related evoked potentials with cardiac potentials evoked long-latency, event-related and showed no link between the central nervous system and autonomic nervous system in relation to some aspects of auditory stimulus processing. Lee and coworkers [11] showed that a continuous five minute white noise of low-to-moderate intensity can evoke a significant response of cardiac autonomic response similar to the startle stimulation. The responses were primarily sympathetic and could be detected through using frequency-domain HRV analysis. It was also observed that the sympathetic activities were significantly correlated with the noise intensities.

Zhao and colleagues investigated the EEG and cardiac autonomic regulation of healthy volunteers during a continuous simulated driving task for 90 minutes [30]. The authors reported that the relative power spectra of different EEG rhythms, the amplitude of P300, the non-linear analysis of heart rate variability and power spectral density of HRV showed statistically significant between differences before and after long-term driving. This study that showed simultaneous responses of heart rate variability (linear and non-linear indices) and the P300, indicating a relationship between those methods of analysis.

Our study presents some points that are worth to be pointed. Although some of the selected studies was performed in animals, the mechanisms observed are currently supported, however, it needs additional clarification for improvement in clinical application. We decided to not select all studies linking auditory stimulation and cardiovascular system in order to focus our objective that was to investigate the relationship between central auditory processing mechanism and autonomic regulation of the heart.

## Conclusion

This study review indicated that there is a strong association between central auditory processing mechanism and cardiac autonomic regulation, indicating that there is great part of studies focusing on musical auditory stimulation effects on heart rate variability. Nevertheless, it is not fully understood the interaction between auditory evoked potential and cardiac autonomic regulation. Additional studies are necessary to clarify this mechanism.

## Competing interests

The authors declare that they have no competing interests.

## Authors’ contributions

All authors participated in the revision of the manuscript. All authors determined the design, interpreted the text and drafted the manuscript. All authors read and gave final approval for the version submitted for publication.
